# HDL particle number and size as predictors of cardiovascular disease

**DOI:** 10.3389/fphar.2015.00218

**Published:** 2015-10-05

**Authors:** Anatol Kontush

**Affiliations:** National Institute for Health and Medical Research (INSERM), UMR-ICAN 1166, Pitié-Salpétrière University Hospital, University of Pierre and Marie Curie -Paris 6Paris, France

**Keywords:** high-density lipoprotein, particle number, HDL cholesterol, plasma concentrations, circulating levels, large HDL, cardiovascular risk, cardiovascular disease, atherosclerosis

## Abstract

Previous studies indicate that reduced concentrations of circulating high-density lipoprotein (HDL) particles can be superior to HDL-cholesterol (HDL-C) levels as a predictor of cardiovascular disease. Measurements of HDL particle numbers, therefore, bear a potential for the improved assessment of cardiovascular risk. Furthermore, such measurement can be relevant for the evaluation of novel therapeutic approaches targeting HDL. Modern in-depth analyses of HDL particle profile may further improve evaluation of cardiovascular risk. Although clinical relevance of circulating concentrations of HDL subpopulations to cardiovascular disease remains controversial, the negative relationship between the number of large HDL particles and cardiovascular disease suggests that assessment of HDL particle profile can be clinically useful. Reduced mean HDL size is equally associated with cardiovascular disease in large-scale clinical studies. Since HDL-C is primarily carried in the circulation by large, lipid-rich HDL particles, the inverse relationship between HDL size and cardiovascular risk can be secondary to those established for plasma levels of HDL particles, HDL-C, and large HDL. The epidemiological data thereby suggest that HDL particle number may represent a more relevant therapeutic target as compared to HDL-C.

However, considerable residual risk exists even at high plasma levels of HDL-C, suggesting that other HDL-associated metrics might provide better assessment of the relationship between HDL metabolism and cardiovascular disease. Moreover, a series of recent large-scale HDL-C-raising trials failed to reduce cardiovascular disease, additionally demonstrating limitations of HDL-C as a therapeutic target. Metrics of HDL functionality represent an obvious alternative to HDL-C (Hovingh et al., 2015); their reliable assessment is, however, challenging in the clinical routine, reflecting their limited use at present. Other, than HDL-C, biomarkers of HDL metabolism frequently employed in the clinic include circulating levels of apolipoprotein A-I (apoA-I, the major HDL protein), concentrations of HDL particles, and mean HDL size. Several large-scale trials have systematically evaluated potential clinical value of these biomarkers. The present article reviews data obtained for the associations between cardiovascular risk and circulating concentrations (numbers), and mean size of HDL particles in humans.

## Total Number of HDL Particles

Several studies evaluated the relationship of cardiovascular risk with total number of HDL particles in the circulation (**Table 1**).

**Table 1:** Clinical trials, which evaluated relationships between high density lipoprotein (HDL) particle number and cardiovascular disease.

Study	Sample size	Population	Follow-up, years	Endpoint	Major result	Odds ratio
VA-HIT	364 cases 697 controls	Men with established CHD and low HDL-C randomized to gemfibrozil or placebo	5.1	MI or CHD death	Elevated number of HDL particles predicted reduced risk of CHD	0.71 (CI 0.61–0.81) per 1 SD increment
MRFIT	214 cases 214 controls	Middle-aged men who died or did not die of CHD matched according to age, number of MetS components, and presence of non-fatal CV event during the trial	18	CHD death	Elevated number of HDL particles predicted reduced risk of CHD death	0.50 (CI 0.26–0.96) for the top vs. bottom quartile
EPIC-Norfolk	1003 cases 1885 controls	Apparently healthy men and women who developed or did not develop CAD	6	Fatal or non-fatal CAD event	Elevated number of HDL particles predicted reduced risk of CAD following multiple adjustment	0.50 (CI 0.37–0.66) for the top vs. bottom quartile
HPS	Statin, 10,033 Placebo, 9988	High-risk men and women with CHD randomized to simvastatin or placebo	5.3	MI, stroke, vascular procedures, CV death, hospital admissions for other cardiac events	Elevated number of HDL particles was associated with reduced risk of coronary events following adjustment for LDL particle number	0.89 (CI 0.85–0.93) per 1 SD increment
MESA	5,598	Men and women without CHD at baseline or lipid altering therapy	5.5	CHD events, cIMT	Elevated number of HDL particles was associated with reduced risk of incident CHD events and decreased cIMT following adjustment for low density lipoprotein (LDL) particle number	0.68 (CI, 0.54–0.85) per 1 SD increment
JUPITER	Statin, 5,367 Placebo, 5,519	Men and women free of cardiovascular disease randomized to rosuvastatin (20 mg/day) or placebo	1	First MI, stroke, hospitalization for unstable angina, arterial revascularization, CV death	Elevated on-treatment number of HDL particles was associated with reduced CV risk in subjects randomized to both the statin and placebo	Statin, 0.73 (CI 0.57–0.93) per 1 SD Placebo, 0.81 (CI 0.67–0.97) per 1 SD

*CAD, coronary artery disease; CHD, coronary heart disease; CI, confidence interval; cIMT, carotid intima-media thickness; CV, cardiovascular; MetS, metabolic syndrome; MI, myocardial infarction; SD, standard deviation*.

In a nested, case–control, secondary prevention Veterans Affairs High-Density Lipoprotein Intervention Trial (VA-HIT), elevated number of HDL particles was a significant predictor of reduced risk of coronary heart disease (CHD) in subjects treated with gemfibrozil or placebo (Otvos et al., 2006). Indeed, increment in total HDL particle number of 1 standard deviation (SD) decreased CHD risk by 29% during a 5-year follow-up (*p* = 0.0001). By contrast, circulating HDL-C and apoA-I levels were unable to predict CHD events in this study (risk reduction of 5%, *p* = 0.42 and 9%, *p* = 0.18, respectively).

Similar results were obtained in a primary prevention Multiple Risk Factor Intervention Trial (MRFIT) of CHD mortality in men at high risk of CHD (Kuller et al., 2007). In two groups of middle-aged men who died or did not die of CHD over 18 years of follow-up, elevated total HDL particle number at baseline significantly reduced CHD death by 50% for the comparison between the top and the bottom quartiles. As in the VA-HIT study, levels of HDL-C were not associated with the endpoint (risk reduction of 0% for the same comparison).

In a case–control European Prospective Investigation into Cancer and Nutrition (EPIC)-Norfolk study of apparently healthy men and women, total HDL particle concentration at baseline was significantly diminished in CHD cases relative to controls (El Harchaoui et al., 2009). As a consequence, HDL particle concentrations in the top quartile decreased coronary artery disease (CAD) risk by 50% relative to the bottom quartile following adjustment for plasma apoB and triglyceride levels.

In a randomized Heart Protection Study (HPS) trial of statin and antioxidant therapy in high-risk men and women, increment in total HDL particle number of 1 SD was associated with a significant risk reduction of 11% for major occlusive coronary events after adjustment for low density lipoprotein (LDL) particle number (Parish et al., 2012). Associations between cardiovascular disease and plasma HDL-C and apoA-I levels were slightly weaker, albeit significant (risk reduction of 9% for each of them). Interestingly, inverse associations of other cardiac events assessed in this study with biomarkers of HDL metabolism were only significant for HDL particle number (risk reduction of 16%) but not for HDL-C and apoA-I levels (risk reduction of 6 and 8%, respectively).

In the Multi-Ethnic Study of Atherosclerosis (MESA) which evaluated men and women without baseline CHD or lipid altering therapy, elevated number of HDL particles revealed an inverse association with both incident CHD events (significant risk reduction of 30% per 1 SD increment in the particle number) and carotid intima-media thickness (cIMT; Mackey et al., 2012). These associations were not affected by adjustment for LDL particle number (risk reduction of 32% per 1 SD increment in the particle number). Plasma HDL-C concentrations revealed similar associations with CHD (significant risk reduction of 26%) and cIMT, which were, however, markedly attenuated after adjustment for plasma levels of atherogenic lipoproteins (non-significant risk reduction of 3%).

In a randomized Justification for the Use of statins in Prevention: an Intervention Trial Evaluating Rosuvastatin (JUPITER) which enrolled subjects without cardiovascular disease assigned to either rosuvastatin or placebo, on-treatment HDL particle number was significantly associated with a composite cardiovascular endpoint in subjects randomized to the statin (risk reduction of 27% per 1 SD following adjustment for traditional cardiovascular risk factors; Mora et al., 2013). Similar associations evaluated for circulating HDL-C and apoA-I levels were not significant (risk reductions of 18 and 14%, respectively). In subjects randomized to placebo, on-treatment concentrations of HDL particles, HDL-C, and apoA-I revealed similar inverse association with the endpoint (significant risk reductions of 19, 25, and 21%, respectively). Interestingly, the association between on-treatment HDL particle numbers and cardiovascular endpoint remained significant after adjustment for HDL-C levels in this study.

## Particle Numbers of Large vs. Small HDL

HDL particles are highly heterogeneous in structure, composition, metabolism, and function. Distinct HDL subpopulations might, therefore, exert differential effects on atherosclerosis (Camont et al., 2011). As a consequence, plasma concentrations of specific HDL subpopulations can be hypothesized to reveal stronger associations with cardiovascular risk as compared to total HDL particle number.

NMR measurements allow distinguishing between large (size 9.4–14.0 nm), medium (8.3–9.3 nm), and small (7.3–8.2 nm) HDL particles (Otvos, 2002). Among these HDL subpopulations, levels of large HDL frequently display inverse relationships with cardiovascular risk in univariate analyses, whereas concentrations of small HDL particles typically reveal positive correlations with the risk (Kuller et al., 2002; Rosenson et al., 2002; Garvey et al., 2003; Festa et al., 2005; Goff et al., 2005; Kathiresan et al., 2006; Otvos et al., 2006; Mora et al., 2007, 2009; van der Steeg et al., 2008; El Harchaoui et al., 2009). Circulating concentrations of large and small HDL particles as well as those of large HDL particles and total LDL particles as measured by NMR are, however, known to be negatively correlated. Such multiple confounding renders data interpretation controversial, complicating proper assessment of the relative roles of distinct HDL subpopulations in cardioprotection (Goff et al., 2005; Jeyarajah et al., 2006; Mora et al., 2007, 2009; El Harchaoui et al., 2009).

As a comparison, particle concentrations of HDL subpopulations can be assessed by the ion mobility assay which readily separates small (7.7–10.5 nm) and large (10.5–14.5 nm) HDL (Musunuru et al., 2009). Such evaluation reveals comparable associations of small and large particles with cardiovascular risk (hazard ratios per 1 SD of 0.78 and 0.75, respectively; Musunuru et al., 2009).

The inverse relationship between particle number of large HDL and cardiovascular disease is further consistent with data on plasma levels (as mg apoA-I/dl) of HDL subpopulations separated by 2D gel electrophoresis. Indeed, circulating concentrations of large alpha-1 HDL are consistently associated with reduced cardiovascular risk (Asztalos and Schaefer, 2003a,b; Asztalos et al., 2003, 2004; Schaefer and Asztalos, 2007a,b). On the other hand, levels of small HDL particles assessed by this methodology are frequently elevated in CHD patients (Asztalos and Schaefer, 2003a,b; Asztalos et al., 2003; Lamon-Fava et al., 2008). Importantly, the power of large alpha-1 HDL to predict CHD risk is typically superior to that of HDL-C in these studies (Asztalos et al., 2003, 2005; Lamon-Fava et al., 2008).

## Mean HDL Size

Mean HDL size can be regarded as an integrative measure of HDL heterogeneity and particle profile. This metric of HDL metabolism can be assessed by both NMR and ion mobility measurements and is capable of predicting cardiovascular risk (Musunuru et al., 2009). An association between mean HDL size and cardiovascular risk is typically inverse. When HDL size is used to predict incident cardiovascular disease, hazard ratios obtained are comparable to those calculated using HDL-C (Mora et al., 2009) and can be predominantly accounted for by traditional cardiovascular risk factors (Arsenault et al., 2009). Consistent with this conclusion, adjustment for circulating concentrations of apoB and triglycerides abolished the association between mean HDL size and CAD risk in the EPIC-Norfolk study, despite reduction in this parameter in cases relative to controls (El Harchaoui et al., 2009).

Interestingly, ion mobility measurements allow distinguishing between two independent components of the association between HDL size and cardiovascular disease (Musunuru et al., 2009). The first component appears to represent an atherogenic lipoprotein phenotype, which includes increased concentrations of triglycerides and small LDL, whereas the second component is related to elevated levels of small HDL particles.

Intriguingly, very high mean HDL size can be paradoxically associated with elevated cardiovascular risk as observed in the EPIC-Norfolk study after multiple adjustment (van der Steeg et al., 2008); this association resembles those reported between cardiovascular disease and very high levels of HDL-C as observed, for example, in the IDEAL study (van der Steeg et al., 2008).

## Effects of Therapeutic Interventions on HDL Particle Concentrations

Therapeutic interventions aimed at increasing HDL-C levels, correcting atherogenic dyslipidemia, and reducing cardiovascular risk may markedly impact HDL particle concentrations. Thus, both niacin (Kuvin et al., 2006; Le et al., 2013) and cholesteryl ester transfer protein (CETP) inhibitors (Brousseau et al., 2005; Ballantyne et al., 2012; Krauss et al., 2012) potently raise HDL-C and preferentially increase circulating levels of large HDL, augmenting mean HDL size. By contrast, effects of niacin and CETP inhibitors on total number of HDL particles are weaker, consistent with their weaker effects on plasma concentrations of apoA-I.

Treatment with statins appears to result in comparable elevations in HDL-C and total HDL particle number (Rosenson et al., 2009). On the other hand, HDL-C-raising effects of fibrates are typically accompanied by preferential elevation of circulating levels of small HDL (Otvos et al., 2006).

## Concluding Remarks

Available data reveal that diminished HDL particle number can be superior to reduced HDL-C levels in terms of cardiovascular risk prediction. This conclusion is supported by the results of the large-scale VA-HIT, MRFIT, HPS, MESA, and JUPITER trials. Measurements of circulating concentrations of HDL particles can, therefore, be useful to improve clinical assessment of cardiovascular risk. This approach can also contribute to the evaluation of novel HDL-targeted therapies, which include CETP inhibitors, peroxisome proliferator-activated receptor (PPAR) activators, and reconstituted HDL.

Further improvement in the evaluation of cardiovascular risk can be provided by in-depth analyses of the HDL particle profile (Mallol et al., 2015; Matyus et al., 2015). Although clinical relevance of circulating concentrations of HDL subpopulations needs to be firmly established (Rosenson et al., 2011), the negative association between the number of large HDL particles and cardiovascular risk suggests that clinical evaluation of the HDL particle profile can be useful.

Diminished mean HDL size represents another biomarker of HDL metabolism associated with cardiovascular disease in large-scale clinical studies. However, available data indicate that this relationship is secondary to those established for plasma levels of HDL particles, HDL-C and large HDL; indeed, HDL-C is primarily carried in the circulation by large, buoyant, lipid-rich HDL.

The epidemiological data thereby suggest that HDL particle number can be superior to HDL-C as a therapeutic target. This conclusion is consistent with the capacity of all HDL particles to exert a variety of atheroprotective biological activities, reflecting a wide spectrum of molecular protein, and lipid species present in HDL (Davidson et al., 2009; Heinecke, 2009; Gordon et al., 2010a,b; Camont et al., 2013; Shah et al., 2013). High compositional heterogeneity of HDL directly results in functional heterogeneity among HDL particles, with small, dense, and protein-rich HDL typically displaying potent biological activities (Kontush and Chapman, 2006a; Camont et al., 2011, 2013). The relative importance of different biological activities of HDL for atheroprotection remains presently unclear, raising a question of the proper evaluation of HDL function *in vitro*. Measurement of any individual HDL activity will obviously provide only limited information on all the aspects of the functionality of the circulating HDL pool. Evaluation of total number of circulating HDL particles may provide a surrogate biomarker reflecting anti-atherogenic HDL function, assuming a proportional association between the two. This hypothesis will require rigorous assessment in clinical and epidemiological studies of HDL-targeted therapies, which have failed to provide unequivocal data in this regard up to now.

## Conflict of Interest Statement

The authors declare that the research was conducted in the absence of any commercial or financial relationships that could be construed as a potential conflict of interest.

## References

[B1] Arsenault B. J. Lemieux I. Despres J. P. Gagnon P. Wareham N. J. Stroes E. S. (2009). HDL particle size and the risk of coronary heart disease in apparently healthy men and women: the EPIC-Norfolk prospective population study. *Atherosclerosis* 206 276–281. 10.1016/j.atherosclerosis.2009.01.044 19268944

[B2] Asztalos B. F. Batista M. Horvath K. V. Cox C. E. Dallal G. E. Morse J. S. (2003). Change in alpha1 HDL concentration predicts progression in coronary artery stenosis. *Arterioscler. Thromb. Vasc. Biol.* 23 847–852. 10.1161/01.ATV.0000066133.32063.BB 12637338

[B3] Asztalos B. F. Collins D. Cupples L. A. Demissie S. Horvath K. V. Bloomfield H. E. (2005). Value of high-density lipoprotein (HDL) subpopulations in predicting recurrent cardiovascular events in the veterans affairs HDL intervention trial. *Arterioscler. Thromb. Vasc. Biol.* 25 2185–2191. 10.1161/01.ATV.0000183727.90611.4f 16123324

[B4] Asztalos B. F. Cupples L. A. Demissie S. Horvath K. V. Cox C. E. Batista M. C. (2004). High-density lipoprotein subpopulation profile and coronary heart disease prevalence in male participants of the Framingham Offspring Study. *Arterioscler. Thromb. Vasc. Biol.* 24 2181–2187. 10.1161/01.ATV.0000146325.93749.a8 15388521

[B5] Asztalos B. F. Schaefer E. J. (2003a). HDL in atherosclerosis: actor or bystander? *Atheroscler. Suppl.* 4 21–29. 10.1016/S1567-5688(03)00006-0 12714034

[B6] Asztalos B. F. Schaefer E. J. (2003b). High-density lipoprotein subpopulations in pathologic conditions. *Am. J. Cardiol.* 91 12E–17E. 10.1016/S0002-9149(02)03383-0 12679198

[B7] Ballantyne C. M. Miller M. Niesor E. J. Burgess T. Kallend D. Stein E. A. (2012). Effect of dalcetrapib plus pravastatin on lipoprotein metabolism and high-density lipoprotein composition and function in dyslipidemic patients: results of a phase IIb dose-ranging study. *Am. Heart. J.* 163 515–521:e1–e3. 10.1016/j.ahj.2011.11.017 22424025

[B8] Brousseau M. E. Diffenderfer M. R. Millar J. S. Nartsupha C. Asztalos B. F. Welty F. K. (2005). Effects of cholesteryl ester transfer protein inhibition on high-density lipoprotein subspecies. Apolipoprotein A-I metabolism, and fecal sterol excretion. *Arterioscler. Thromb. Vasc. Biol.* 25 1057–1064. 15761191 10.1161/01.ATV.0000161928.16334.dd PMC3229922

[B9] Camont L. Chapman M. J. Kontush A. (2011). Biological activities of HDL subpopulations and their relevance to cardiovascular disease. *Trends Mol. Med.* 17 594–603. 10.1016/j.molmed.2011.05.013 21839683

[B10] Camont L. Lhomme M. Rached F. Le Goff W. Negre-Salvayre A. Salvayre R. (2013). Small, dense high-density lipoprotein-3 particles are enriched in negatively charged phospholipids: relevance to cellular cholesterol eﬄux, antioxidative, antithrombotic, anti-inflammatory, and antiapoptotic functionalities. *Arterioscler. Thromb. Vasc. Biol.* 33 2715–2723. 10.1161/ATVBAHA.113.301468 24092747

[B11] Davidson W. S. Silva R. A. Chantepie S. Lagor W. R. Chapman M. J. Kontush A. (2009). Proteomic analysis of defined HDL subpopulations reveals particle-specific protein clusters: relevance to antioxidative function. *Arterioscler. Thromb. Vasc. Biol.* 29 870–876. 10.1161/ATVBAHA.109.186031 19325143 PMC2845307

[B12] El Harchaoui K. Arsenault B. J. Franssen R. Despres J. P. Hovingh G. K. Stroes E. S. (2009). High-density lipoprotein particle size and concentration and coronary risk. *Ann. Intern. Med.* 150 84–93. 10.7326/0003-4819-150-2-200901200-00006 19153411

[B13] Festa A. Williams K. Hanley A. J. G. Otvos J. D. Goff D. C. Wagenknecht L. E. (2005). Nuclear magnetic resonance lipoprotein abnormalities in prediabetic subjects in the insulin resistance atherosclerosis study. *Circulation* 111 3465–3472. 10.1161/CIRCULATIONAHA.104.512079 15983261

[B14] Garvey W. T. Kwon S. Zheng D. Shaughnessy S. Wallace P. Hutto A. (2003). Effects of insulin resistance and type 2 diabetes on lipoprotein subclass particle size and concentration determined by nuclear magnetic resonance. *Diabetes Metab. Res. Rev.* 52 453–462. 10.2337/diabetes.52.2.453 12540621

[B15] Goff D. C. Jr. D’Agostino R. B. Jr. Haffner S. M. Otvos J. D. (2005). Insulin resistance and adiposity influence lipoprotein size and subclass concentrations. Results from the insulin resistance atherosclerosis study. *Metabolism* 54 264–270. 15690322 10.1016/j.metabol.2004.09.002

[B16] Gordon S. Durairaj A. Lu J. L. Davidson W. S. (2010a). High-density lipoprotein proteomics: identifying new drug targets and biomarkers by understanding functionality. *Curr. Cardiovasc. Risk Rep.* 4 1–8. 10.1007/s12170-009-0069-9 20625533 PMC2901108

[B17] Gordon S. M. Deng J. Lu L. J. Davidson W. S. (2010b). Proteomic characterization of human plasma high density lipoprotein fractionated by gel filtration chromatography. *J. Proteome Res.* 9 5239–5249. 10.1021/pr100520x 20718489 PMC3100175

[B18] Heinecke J. W. (2009). The HDL proteome: a marker - and perhaps mediator - of coronary artery disease. *J. Lipid Res.* 50 S167–S171. 10.1194/jlr.R800097-JLR200 19060251 PMC2674735

[B19] Hovingh G. K. Rader D. J. Hegele R. A. (2015). HDL re-examined. *Curr. Opin. Lipidol.* 26 127–132. 10.1097/MOL.0000000000000161 25692348

[B20] Jeyarajah E. J. Cromwell W. C. Otvos J. D. (2006). Lipoprotein particle analysis by nuclear magnetic resonance spectroscopy. *Clin. Lab. Med.* 26 847–870. 10.1016/j.cll.2006.07.006 17110242

[B21] Kathiresan S. Otvos J. D. Sullivan L. M. Keyes M. J. Schaefer E. J. Wilson P. W. (2006). Increased small low-density lipoprotein particle number: a prominent feature of the metabolic syndrome in the Framingham Heart Study. *Circulation* 113 20–29. 10.1161/CIRCULATIONAHA.105.567107 16380547

[B22] Kontush A. Chapman M. J. (2006a). Antiatherogenic small, dense HDL - guardian angel of the arterial wall? *Nat. Clin. Pract. Cardiovasc. Med.* 3 144–153. 10.1038/ncpcardio0500 16505860

[B23] Kontush A. Chapman M. J. (2006b). Functionally defective HDL: a new therapeutic target at the crossroads of dyslipidemia, inflammation and atherosclerosis. *Pharmacol. Rev.* 3 342–374. 10.1124/pr.58.3.1 16968945

[B24] Krauss R. M. Wojnooski K. Orr J. Geaney J. C. Pinto C. A. Liu Y. (2012). Changes in lipoprotein subfraction concentration and composition in healthy individuals treated with the CETP inhibitor anacetrapib. *J. Lipid Res.* 53 540–547. 10.1194/jlr.M018010 22180633 PMC3276477

[B25] Kuller L. Arnold A. Tracy R. Otvos J. Burke G. Psaty B. (2002). Nuclear magnetic resonance spectroscopy of lipoproteins and risk of coronary heart disease in the Cardiovascular Health Study. *Arterioscler. Thromb. Vasc. Biol.* 22 1175–1180. 10.1161/01.ATV.0000022015.97341.3A 12117734

[B26] Kuller L. H. Grandits G. Cohen J. D. Neaton J. D. Prineas R. Multiple Risk Factor Intervention Trial Research Group (2007). Lipoprotein particles, insulin, adiponectin, C-reactive protein and risk of coronary heart disease among men with metabolic syndrome. *Atherosclerosis* 195 122–128. 17011566 10.1016/j.atherosclerosis.2006.09.001 PMC2098784

[B27] Kuvin J. T. Dave D. M. Sliney K. A. Mooney P. Patel A. R. Kimmelstiel C. D. (2006). Effects of extended-release niacin on lipoprotein particle size, distribution, and inflammatory markers in patients with coronary artery disease. *Am. J. Cardiol.* 98 743–745. 10.1016/j.amjcard.2006.04.011 16950175

[B28] Lamon-Fava S. Herrington D. M. Reboussin D. M. Sherman M. Horvath K. V. Cupples L. A. (2008). Plasma levels of HDL subpopulations and remnant lipoproteins predict the extent of angiographically-defined coronary artery disease in postmenopausal women. *Arterioscler. Thromb. Vasc. Biol.* 28 575–579. 10.1161/ATVBAHA.107.157123 18174456

[B29] Le N. A. Jin R. Tomassini J. E. Tershakovec A. M. Neff D. R. Wilson P. W. (2013). Changes in lipoprotein particle number with ezetimibe/simvastatin coadministered with extended-release niacin in hyperlipidemic patients. *J. Am. Heart. Assoc.* 2:e000037 10.1161/JAHA.113.000037 PMC3828803 23926117

[B30] Mackey R. H. Greenland P. Goff D. C. Jr. Lloyd-Jones D. Sibley C. T. Mora S. (2012). High-density lipoprotein cholesterol and particle concentrations, carotid atherosclerosis, and coronary events: MESA (Multi-Ethnic Study of Atherosclerosis). *J. Am. Coll. Cardiol.* 60 508–516. 10.1016/j.jacc.2012.03.060 22796256 PMC3411890

[B31] Mallol R. Amigo N. Rodriguez M. A. Heras M. Vinaixa M. Plana N. (2015). Liposcale: a novel advanced lipoprotein test based on 2D diffusion-ordered 1H NMR spectroscopy. *J. Lipid Res.* 56 737–746. 10.1194/jlr.D050120 25568061 PMC4340320

[B32] Matyus S. P. Braun P. J. Wolak-Dinsmore J. Saenger A. K. Jeyarajah E. J. Shalaurova I. (2015). HDL particle number measured on the Vantera(R), the first clinical NMR analyzer. *Clin. Biochem.* 48 148–155. 10.1016/j.clinbiochem.2014.11.017 25438074

[B33] Mora S. Glynn R. J. Ridker P. M. (2013). High-density lipoprotein cholesterol, size, particle number, and residual vascular risk after potent statin therapy. *Circulation* 128 1189–1197. 10.1161/CIRCULATIONAHA.113.002671 24002795 PMC3807967

[B34] Mora S. Otvos J. D. Rifai N. Rosenson R. S. Buring J. E. Ridker P. M. (2009). Lipoprotein particle profiles by nuclear magnetic resonance compared with standard lipids and apolipoproteins in predicting incident cardiovascular disease in women. *Circulation* 119 931–939. 10.1161/CIRCULATIONAHA.108.816181 19204302 PMC2663974

[B35] Mora S. Szklo M. Otvos J. D. Greenland P. Psaty B. M. Goff D. C. (2007). LDL particle subclasses. LDL particle size, and carotid atherosclerosis in the Multi-Ethnic Study of Atherosclerosis (MESA). *Atherosclerosis* 192 211–217. 16765964 10.1016/j.atherosclerosis.2006.05.007

[B36] Musunuru K. Orho-Melander M. Caulfield M. P. Li S. Salameh W. A. Reitz R. E. (2009). Ion mobility analysis of lipoprotein subfractions identifies three independent axes of cardiovascular risk. *Arterioscler. Thromb. Vasc. Biol.* 29 1975–1980. 10.1161/ATVBAHA.109.190405 19729614 PMC2772123

[B37] Otvos J. D. (2002). Measurement of lipoprotein subclass profiles by nuclear magnetic resonance spectroscopy. *Clin. Lab.* 48 171–180. 11934219

[B38] Otvos J. D. Collins D. Freedman D. S. Shalaurova I. Schaefer E. J. Mcnamara J. R. (2006). Low-density lipoprotein and high-density lipoprotein particle subclasses predict coronary events and are favorably changed by gemfibrozil therapy in the Veterans Affairs High-Density Lipoprotein Intervention Trial. *Circulation* 113 1556–1563. 10.1161/CIRCULATIONAHA.105.565135 16534013

[B39] Parish S. Offer A. Clarke R. Hopewell J. C. Hill M. R. Otvos J. D. (2012). Lipids and lipoproteins and risk of different vascular events in the MRC/BHF Heart Protection Study. *Circulation* 125 2469–2478. 10.1161/CIRCULATIONAHA.111.073684 22539783

[B40] Riwanto M. Landmesser U. (2013). High density lipoproteins and endothelial functions: mechanistic insights and alterations in cardiovascular disease. *J. Lipid Res.* 54 3227–3243. 10.1194/jlr.R037762 23873269 PMC3826672

[B41] Rosenson R. S. Brewer H. B. Jr. Chapman M. J. Fazio S. Hussain M. M. Kontush A. (2011). HDL Measures, Particle Heterogeneity, Proposed Nomenclature, and Relation to Atherosclerotic Cardiovascular Events. *Clin. Chem.* 57 392–410. 10.1373/clinchem.2010.155333 21266551

[B42] Rosenson R. S. Otvos J. D. Freedman D. S. (2002). Relations of lipoprotein subclass levels and low-density lipoprotein size to progression of coronary artery disease in the Pravastatin Limitation of Atherosclerosis in the Coronary Arteries (PLAC-I) trial. *Am. J. Cardiol.* 90 89–94. 10.1016/S0002-9149(02)02427-X 12106834

[B43] Rosenson R. S. Otvos J. D. Hsia J. (2009). Effects of rosuvastatin and atorvastatin on LDL and HDL particle concentrations in patients with metabolic syndrome: a randomized, double-blind, controlled study. *Diabetes Care* 32 1087–1091. 10.2337/dc08-1681 19265025 PMC2681027

[B44] Schaefer E. J. Asztalos B. F. (2007a). Increasing high-density lipoprotein cholesterol, inhibition of cholesteryl ester transfer protein, and heart disease risk reduction. *Am. J. Cardiol.* 100 S25–S31. 10.1016/j.amjcard.2007.08.010 18047849

[B45] Schaefer E. J. Asztalos B. F. (2007b). Where are we with high-density lipoprotein raising and inhibition of cholesteryl ester transfer for heart disease risk reduction? *Curr. Opin. Cardiol.* 22 373–378. 10.1097/HCO.0b013e3281fbd3c7 17556892

[B46] Shah A. S. Tan L. Long J. L. Davidson W. S. (2013). Proteomic diversity of high density lipoproteins: our emerging understanding of its importance in lipid transport and beyond. *J. Lipid Res.* 54 2575–2585. 10.1194/jlr.R035725 23434634 PMC3770071

[B47] van der Steeg W. A. Holme I. Boekholdt S. M. Larsen M. L. Lindahl C. Stroes E. S. (2008). High-density lipoprotein cholesterol, high-density lipoprotein particle size, and apolipoprotein A-I: significance for cardiovascular risk: the IDEAL and EPIC-Norfolk studies. *J. Am. Coll. Cardiol.* 51 634–642. 10.1016/j.jacc.2007.09.060 18261682

